# Human Diseases from Wildlife by Michael R. Conover and Rosanna M. Vail

**DOI:** 10.4269/ajtmh.15-0558

**Published:** 2016-01-06

**Authors:** S. Jack

**Affiliations:** Mississippi State University PO Box 9825 Mississippi State, Mississippi 39762 E-mail: jack@cvm.msstate.edu

***Human Diseases from Wildlife.*** Michael R. Conover, and Rosanna M. Vail. ISBN: 978-1-4665-6214-1

Michael Conover and Rosanna Vail are to be commended for compiling the information in Human Diseases from Wildlife, CRC Press (ISBN: 978-1-4665-6214-1). In the true spirit of One Health, this book will prove to be an asset in many collections. The book is 527 pages, including four appendices and a lengthy topical index. Following an introductory chapter, the text is divided into seven sections based on disease-causing agents (bacteria, sprirochetes, *Rickettsia*, viruses, fungi, prions, and parasites). There are then multiple chapters in each section to address specific diseases, 30 in total. Each chapter follows a set pattern of topics (introduction/history, symptoms in humans, infection in animals, how humans contract the disease, medical treatment, how to reduce human risk, eradication of the disease from a country, and literature cited). This format provides a simple and easy to follow reference for each topic. The appendices address medical definitions, scientific names of animal species, photos of North American vectors and disease they transmit, and a list of zoonotic diseases covered in this book.

As noted, this book provides a quick reference to field biologists, students, and health care providers to the basics of each of the extensive list of diseases. This book is largely focused, although not exclusively, upon North American wildlife. It is not extremely detailed, but provides basic information, and will be an ideal “first stop” reference. Since the book is organized by agents and not syndromes or manifestations, readers will need to know what disease they wish to review. One of the highlights of this text are the multiple “sidebars” found within each chapter. These provide anecdotal stories about the various diseases and their recent presentations and relevance/impact upon people and animals. Also, each chapter begins with one or several interesting quotes concerning the diseases. A disappointment in the book is the images. All are black and white and not of the highest quality. Most figures and tables significantly add to the textual presentations. Although the bacterial and viral disease chapters are quite extensive, the coverage of fungal diseases (only *Cryptococcus* and *Histoplasma*) is limited. For parasites, only baylisascariasis, trichinellosis, and swimmers' itch/giardiasis are addressed.

All-in-all, I am pleased to have this book in my collection. It will be an asset for One health-related courses, and serve as a ready reference for students wishing to know more about zoonotic diseases associated with wildlife.

